# Balloon dilatation method to assist release of the snare trapped in a gastric bezoar

**DOI:** 10.1055/a-2641-1923

**Published:** 2025-08-14

**Authors:** Xubiao Nie, Ying Zuo, Jiao Xie, Yanhong Wang, Changjiang Hu, Chaoqiang Fan

**Affiliations:** 1 105785 Department of Gastroenterology, Second Affiliated Hospital, Army Medical University, Chongqing, China

A 70-year-old male patient presented with a two-month history of upper abdominal pain. Two months before admission, he experienced discomfort in the upper abdomen following the ingestion of four persimmons on an empty stomach. Despite receiving intravenous fluids and oral medications, his symptoms persisted without significant improvement. He was subsequently referred to our hospital for further evaluation and management. Gastroscopy confirmed the presence of a gastric bezoar. We endeavored to extract the bezoar endoscopically; however, during the procedure, the snare was unable to fully incise the bezoar. Instead, the snare became embedded within it and could not be dislodged. We then inserted another endoscope through the mouth, utilized a dilation balloon to expand the snare, and adjusted its orientation to successfully release it ( Video 1 ). Following this procedure, we recommended that the patient continue oral administration of cola and sodium bicarbonate solution to soften the bezoar and schedule a follow-up endoscopic intervention for definitive removal.

Balloon dilation to release a snare trapped in a gastric bezoar.Video 1

The treatment of gastric bezoars predominantly relies on the use of snares 1 2 , but there is a certain probability of the snare being trapped in a gastric bezoar. For managing a snare entrapped in a gastric bezoar, previously reported methods include the clamping and turning technique using foreign body forceps 3 . We successfully released a snare trapped in a gastric bezoar utilizing a balloon dilation-assisted method, thereby avoiding surgical intervention.

Endoscopy_UCTN_Code_CPL_1AH_2AK

## Notes

Endoscopy E-Videos https://eref.thieme.de/e-videos
*E-Videos* is an open access online section of the journal *Endoscopy* , reporting on interesting cases and new techniques in gastroenterological endoscopy. All papers include a high-quality video and are published with a Creative Commons CC-BY license. Endoscopy E-Videos qualify for HINARI discounts and waivers and eligibility is automatically checked during the submission process. We grant 100% waivers to articles whose corresponding authors are based in Group A countries and 50% waivers to those who are based in Group B countries as classified by Research4Life (see: https://www.research4life.org/access/eligibility/ ). This section has its own submission website at https://mc.manuscriptcentral.com/e-videos .
